# Collaborative evaluation of a pilot involvement opportunity: Cochrane Common Mental Disorders Voice of Experience College

**DOI:** 10.1111/hex.13835

**Published:** 2023-08-15

**Authors:** Sarah Knowles, Karen Morley, Rob Foster, Amy Middleton, Semra Pinar, Fiona Rose, Emma Williams, Jessica Hendon, Rachel Churchill

**Affiliations:** ^1^ Centre for Reviews and Dissemination University of York York UK; ^2^ Cochrane Common Mental Disorders Group, Centre for Reviews and Dissemination University of York York UK; ^3^ Voice of Experience College, Centre for Reviews and Dissemination University of York York UK

**Keywords:** evidence synthesis, mental health, patient and public involvement, patient capacity building, systematic reviews

## Abstract

**Background:**

Involving consumers in systematic reviews can make them more valuable and help achieve goals around transparency. Systematic reviews are technically complex and training can be needed to enable consumers to engage with them fully. The Cochrane Common Mental Disorders group sought to engage people with lived experience of mental health problems in the Voice of Experience College, three workshops introducing them to systematic review methods and to opportunities to contribute as Cochrane consumers. We aimed to collectively evaluate the College from the perspective of both facilitators and consumers, to critically reflect on the experience, and to identify how the College could be sustained and spread to other review groups.

**Methods:**

This study was a longitudinal qualitative and collaborative evaluation, structured around normalisation process theory. Both facilitators and consumers were involved in not only providing their perspectives but also reflecting on these together to identify key learning points.

**Results:**

The workshops were positively evaluated as being engaging and supportive, largely due to the relational skills of the facilitators, and their willingness to engage in joint or two‐way learning. The College suffered from a lack of clarity over the role of consumers after the College itself, with a need for greater communication to check assumptions and clarify expectations. This was not achieved due to pandemic disruptions, which nevertheless demonstrated that resources for involvement were not prioritised as core business during this period.

**Conclusions:**

Soft skills around communication and support are crucial to effective consumer engagement. Sustaining involvement requires sustained communication and opportunities to reflect together on opportunities and challenges. This requires committed resources to ensure involvement activity is prioritised. This is critical as negative experiences later in the involvement journey can undermine originally positive experiences if contributors are unclear as to what their involvement can lead to. Open discussions about this are necessary to avoid conflicting assumptions. The spread of the approach to other review groups could be achieved by flexibly adapting to group‐specific resources and settings, but maintaining a core focus on collaborative relationships as the key mechanism of engagement.

**Patient and Public Contribution:**

Public contributors were collaborators throughout the evaluation process and have co‐authored the paper.

## 1 BACKGROUND

Systematic reviews are complex pieces of research used to inform health care decision‐making. Using systematic reviews to inform health care decisions often requires an understanding of multiple design, analysis and reporting issues. Cochrane is the largest international producer of systematic reviews (https://www.cochranelibrary.com/). Historically, Cochrane's focus has been on the development of ‘gold standard' methods to produce systematic reviews.^1^ However, in recent years Cochrane has broadened its remit to include a commitment to helping translate knowledge from Cochrane reviews into practice (https://www.cochrane.org/about-us/strategy-to-2020). A crucial audience for this work is patients and the public themselves.^2^ It is argued that the involvement of patients and members of the public with lived experience of illness can make reviews more valuable and accurate, as well as improve transparency in review production.^3^ To make Cochrane review findings accessible and valuable to the public and to support them to make shared decisions about their health, Cochrane recognises that such users need to meaningfully contribute to the production of reviews. To support this endeavour, Cochrane provides tailored training to provide skills and knowledge to help interested volunteers to engage with Cochrane reviews and their production and to meaningfully contribute their own knowledge and experience (https://training.cochrane.org/essentials). Cochrane now has a vibrant community of Cochrane consumers who continue to grow in numbers (where ‘Consumers’ is the preferred term within Cochrane to describe patients, carers and family members with first‐hand experience of a health care condition).*

In 2018, the Cochrane Common Mental Disorders (CCMD) Review Group identified a need to extend its local community of ‘consumers’ to help provide more patient and public involvement (PPI) input into the work of the Group. To extend its consumer involvement, in 2019, CCMD initiated work with Cochrane colleagues to develop a collaborative involvement opportunity. An introductory learning opportunity was developed for a cohort of consumers to attend a series of three workshops about evidence synthesis. CCMD hoped that the opportunity would act as a gateway into the wider work of the Group (https://cmd.cochrane.org/about-us/involvement/voice-experience-college-201920) and improve CCMDs' PPI provision. It was called ‘the Voice of Experience College’ (VoE College). The college would also provide facilitated access to the online learning modules, providing an opportunity for beneficial individual learning.

If and how contributors should gain learning in research methods or approaches can be a contentious issue. In the literature on co‐production, some researchers have expressed concerns that training can involve professionalisation of lived experience contributors, which compromises the external nature of their contribution^4^ or can be a way of making contributors conform to academic processes.^5^ It has been argued, however, that such learning can be necessary and appropriate when contributors are expected to become involved with technically demanding topics or perform specialist activities (e.g., contributing to qualitative analysis, to working as trainers themselves or consulting on methodologically complex research topics such as data linkage^6^, ^7^, ^8^). Most importantly, binary perceptions of ‘lay’ and ‘expert’ can be perceived as simplistic and patronising by contributors themselves, who perceive training as valuable^9^ and as crucial to, rather than undermining, their ability to bring lay knowledge into the process. The potential for authentic contributions, meaning those which have an impact on research and researchers, can be enhanced if contributors have greater awareness, familiarity and confidence to engage with new academic concepts and materials.^10^

Similar to the contention around training, involvement evaluation has itself been criticised for potentially imposing researchers' concerns and understanding onto the process, meaning involvement is evaluated instrumentally in terms of whether or not it meets the researchers' needs.^11^ Evaluation can potentially undermine the equal importance of lived experience expertise by implying it needs to be justified as effective, with the observation that the involvement of other professionals in research (e.g., from other disciplines) is not subject to evaluation in the same way.^12^

We observe in both these issues that the concern focuses on the neglect of contributor experience and relative privileging of researcher perspectives, with both training and evaluation seen as occurring to help researchers, rather than being with and for contributors themselves. We therefore explicitly aimed for the evaluation process to be collaborative with contributors, to ensure that we were not only seeking their feedback but working with them as partners to reflect on the process and impacts of the College, mirroring the intended ethos of the College itself. This paper provides a joint reflection from both contributors and CCMD facilitators. (In the remainder of the paper, ‘CCMD facilitators refers to the CCMD Managing Editor [J. H.] and Cochrane consumer [K. M.], who, alongside the Cochrane Consumer Engagement Officer and Cochrane Learning Manager, were responsible for the design and delivery of the College).

Evaluation and reporting of research involvement have tended to be atheoretical.^13^ In this evaluation, the researcher lead author suggested using an implementation science framework, normalisation process theory (NPT) to guide the evaluation. NPT has been successfully applied previously to help understand challenges to involvement in research.^9^, ^14^, ^15^ We saw NPT as being helpful in bringing a multifactorial perspective to consider the pragmatic question of how the College worked, or struggled to work, in practice and to consider the activities and attitudes necessary to support both initial engagement with the College and support future collaboration. CCMD was interested in how to encourage the involvement of members of the group and sustain involvement after the VoE College had been completed, and how to spread the learning about engaging contributors in gaining knowledge of reviews to other groups in Cochrane, and a theoretical framework was considered helpful for contextualising findings relevant to sustainability and scale of the approach. The framework was introduced to the contributors as an option for supporting the evaluation, and a collective decision was made to use the framework. A qualitative and participatory approach to evaluation was not only necessary within our collaborative ethos, but also appropriate to evaluating the College as a collaboration, as analysis of changes in perceived capacity or comparison to baselines of research knowledge can fail to align with the collaborative dynamics of involvement.^16^

For CCMD, the primary aim of the VoE College workshops was to equip people with new skills and knowledge that would be helpful to them if they then chose to become more involved in the work of Cochrane and in particular CCMD.^17^ This paper reports on the evaluation of the College from the perspective of the CCMD facilitators and the contributors who attended.

### 1.1 Aim

The aim of this study was to collaboratively, with the CCMD facilitators and contributors, evaluate the VoE College.

### 1.2 Research questions (RQs)

1. What do the CCMD facilitators and public contributors involved consider to be the successes and challenges of the VoE College?
2. What would help support the sustainability and spread of this approach?

## 2 METHOD

This study was a longitudinal qualitative collaborative evaluation.

### 2.1 Setting

In brief, the VoE College involved three interactive face‐to‐face workshops (held at the University of York; Table 1) and each workshop followed a planned programme of activities^18^ (Supporting Information: Appendix 1). The workshops included a combination of presentations and interactive group activities such as group examination of misleading health claims in the media, mock clinical trials and role‐play exercises.

**Table 1:** Workshops and attendees.

Workshop	Date	Topic	Number of contributors
1	September 2019	Discovery	14
2	November 2019	Understanding	10
3	January 2020	Applying	8

The workshops were complemented by optional learning tasks between sessions involving the completion of the Cochrane Evidence Essentials online modules (https://training.cochrane.org/essentials). The programme of workshop activities was designed to enable contributors to develop skills focused around:

1. Understanding and using the evidence produced by Cochrane and other sources.
2. Providing contributors with a technical understanding about systematic reviews and evidence appraisal.
3. Introducing them to the work of Cochrane and helping them understand the role of consumers in improving that work.

Recruitment of contributors (attendees) for the VoE College was done by advertising using social media, local involvement mailing lists and the University of York newsletters. Interested parties were invited to submit contact information via an online form. A face‐to‐face meeting was then offered to help provide contributors with additional information before they made a decision to attend the workshop programme.

The number of contributors diminished over the course of the workshops. The main reasons cited for nonattendance at subsequent workshops were related to a change in personal circumstances during the 5‐month period, rather than a judgement that the College was not right for the individual. Lunch was provided and travel expenses were covered.

All the workshops also involved attendance from the managing editor of CCMD (J. H.), a Cochrane consumer working with CCMD and Cochrane Learning (K. M.) and the Cochrane Consumer Engagement Officer. The Cochrane Learning Manager attended Workshops 1 and 3. Workshop 2 was also attended by an external representative from Tees, Esk and Wear Valleys NHS Foundation Trust Research Team (CCMD Partner NHS Trust) as well as members of the CCMD editorial team who were available to answer questions during the breaks. Workshop 3 was attended by two general practitioners (GPs) who participated in discussions. GP involvement was sourced via their association with CCMD and the Hull York Medical School.

The workshop environment was intended to be informal and collaborative. In Workshop 1 the contributors and delivery team worked together to establish ground rules for working together. These were then turned into an illustrative poster by K. M. and displayed on the wall in the following sessions.

### 2.2 Evaluation feedback

Evaluation feedback was gathered during the VoE College, immediately after the VoE College (Time 1—T1) and 12 months after (Time 2—T2).

*During the VoE College*: All attendees were sent postworkshop feedback surveys after each workshop. The questionnaire asked attendees to rate the level of the material (was it too simple, too challenging or about right), report any concerns about access to the workshops and provided free text comments for general feedback. Ten surveys were completed after Workshop 1, eight after Workshop 2 and five after Workshop 3.

*After the College, T1*: A collaborative reflective meeting was attended by six contributors who were participants in the College, CCMD facilitators who had been involved in designing and delivering the VoE College (J. H., Cochrane Managing Editor, and K. M., Cochrane consumer). The meeting was facilitated by a researcher with experience in evaluating involvement activities (S. K.). For contributors who could not attend, one‐to‐one feedback was sought through a conversation with S. K. Additional reflections from the Cochrane Consumer Engagement Officer before the meeting (during a meeting between S. K., J. H., K. M. and the Officer) were also included as they were unable to attend the meeting itself.

The framework used for the meeting discussion was NPT. The meeting began with an explanation of the framework in lay terms, checking with the contributors whether they felt the constructs were appropriate, seeking agreement on whether to use the framework for further reporting and agreeing that the discussion was not limited to those constructs.

### 2.3 NPT constructs and questions used to guide the discussion

*Why* are we doing this work? ‘Coherence’

*What is the value of the College, why is it needed, what are we wanting to achieve?*

*Who* does the work? ‘Cognitive Participation’

*Who are the people who should be involved, what skills or interests do they need?*

*How* does it work? ‘Collective Action’

*How does it work in practice? What makes it feasible, and what gets in the way?*

*What* did we do that worked well, and how can we do better? ‘Reflexive Monitoring’

*What were the impacts that were important? What changed over time, or should we change in future?*

Participants split into two groups. They were given 10 min to note down their thoughts and responses and discuss the question among their group. Each group then shared their key observations or questions. These were noted on a whiteboard by S. K. This was followed by a whole group discussion of these key points, identifying consensus and considering whether any issues were being missed.

*Twelve‐month follow‐up, T2*: Contributors were invited by email to have a follow‐up discussion with the evaluation lead (S. K.). Interview was done over video teleconferencing or telephone, or text response to the questions via email. A brief discussion guide was designed to revisit the earlier findings, as a form of interim member‐checking, and to reflect on contributions over the past year (Supporting Information: Appendix 2).

*CCMD facilitators (J. H. and K. M.) and the Consumer Engagement Officer*: Two‐hour reflection session as a group was held online using Zoom, with Google Jamboard to capture feedback.

The decision at T2 for the contributors to provide feedback individually and the CCMD facilitators as a group was pragmatic, based on availability, rather than being a deliberate choice to collect feedback in a different format.

### 2.4 Analysis and presentation of feedback

Analysis was collective and concurrent, meaning that the evaluation reflections were a joint process occurring in real‐time through dialogue, as opposed to contributors being viewed as providing discrete feedback, which was separately analysed by members of the Cochrane team. NPT was agreed upon as an organising framework to record and present feedback. We agreed that feedback could be captured to be potentially reported in this paper, to aid in the transparency of the findings, with verbal agreement from meeting attendees and interview respondents that quotes would be anonymised and that all contributors would have the opportunity to review the report and edit or remove any feedback if they wished to do so. Feedback was captured in the reflective meeting in the form of post‐it note comments and group comments captured on a whiteboard. In the one‐to‐one interviews, the interviewer (S. K.) took written notes including capturing quotations. To prepare this paper, S. K. engaged in selective coding of the reflective meeting and interview feedback to provide illustrations of the key learning points. The paper itself was reviewed by all co‐authors, including contributors and CCMD facilitators, to enable a final stage of collective reflection and agreement on the core findings.

#### 2.4.1 Ethical considerations on the evaluation analysis and reporting

In the United Kingdom, where the work was conducted, Health Research Authority guidance states that public involvement in research does not require Research Ethics Committee (REC) approval. This is because REC approval is necessary for research conducted on participants, whereas public involvement involves collaboration between researchers and public contributors, with the latter contributing to the process as experts, rather than participating in a process that is managed or owned by researchers exclusively. We sought guidance from the Chair of the University Health Sciences REC and confirmed with them that the evaluation as a whole was a public involvement activity, rather than a piece of research conducted by researchers on research participants.

We were mindful however that in conducting an evaluation process collaboratively, including gathering feedback in a structured way, conducting analysis together and reporting findings using a pre‐existing theoretical framework, there was potential for these involvement activities to be considered as overlapping with qualitative research. We reflected on Pandya‐Wood et al.'s^19^ ‘ethically conscious standards’ for public involvement in research, which advises researchers not to confuse qualitative research and public involvement activity, as this risks consultation being mistakenly considered appropriate for research ethical approval. The process throughout was described and enacted as a collaboration and shared reflection, with key decisions around reporting and agreement on lessons learned made jointly, and hence we do not consider that research was conducted on or about participants at any point, but rather that we, as a collective group, engaged with each other in a collective evaluation process.

We nevertheless wanted our process to be transparent and thorough for the wider community, and hence report our stages of collecting and analysing feedback, and include quotations from both contributors and Cochrane team members to illustrate how and why we came to our conclusions. We therefore gained verbal consent in both the group meeting and the one‐to‐one follow‐ups for feedback to be recorded and potentially reproduced, once anonymised, in evaluation reporting. Both interim reports (post‐T1) and final reports were shared with all who were involved, with the option to remove or edit feedback if they wished. This overall approach was discussed at the beginning of the evaluation, and all authors (consumers and professionals) agreed on this final representation of the ethical approach taken.

## 3 RESULTS

### 3.1 RQ1. What do the CCMD facilitators and public contributors involved consider to be the successes and challenges of the VoE College?

1. *Survey responses during the study*:

Survey responses indicated approval of the College workshops themselves, in terms of appropriate content (with exercises and materials considered to be pitched at the right level), effective facilitation, and also practical considerations such as accessibility.

I really enjoyed meeting the other participants. Great group of people. Great facilitators. (Survey Workshop 1)

It was welcoming, professionally & warmly presented, and absolutely fascinating. I had my eyes opened and learned a lot. (Survey Workshop 1)

There was some indication however that there was a lack of clarity around the expected role for contributors after the VoE College (i.e., regarding becoming a regular volunteer with Cochrane).

I am still unclear as to the overall purpose of attending the College other than for personal learning … is it hoped that we will volunteer for you after? I'm finding it all really positive, but what are the future plans? (Survey Workshop 2)

Comments after Workshop 3 also indicated greater clarity about the next steps was valued:

It was my favourite workshop! I think because it told me about how to get involved afterwards. (Survey Workshop 3)

2. *Reflective meeting and interviews at T1 and T2*:

We summarise the findings for RQ1 in Table 2, columns 2 and 3, organised according to the NPT constructs. These are illustrated with quotes from both evaluation times, as they include reflections at T2 about whether the original reflections had changed or were the same. Quotations indicate T1 or T2 and whether they came from a member of the CCMD facilitators (F) or contributor (C).

**Table 2:** Reflections on the College at T1 and T2, organised by NPT construct.

	T1 contributors	T1 CCMD facilitators	T2 (both)
Coherence	The College as an opportunity to learn and do something new. ‘It was a new experience, I wanted to use my brain again’. (Meeting—C) An opportunity to contribute something meaningful and be part of something important. ‘I wanted to give something back’. (T2—C3) ‘In the long term, I want to help people’. (Meeting—C)	Wider goals around democratisation of knowledge and demystifying review production. Practically we need consumer reviewers to meet Cochrane requirements. Felt consumer involvement could be extractive, taking the time and experience of consumer reviewers, and wanted there to be something given to them, through opportunities and upskilling. ‘It's an investment in people. We want to understand their needs, their interests’. (T1—F) Welcomed opportunity to learn from the contributors, not just provide learning ‘Cochrane has a lot to learn from stakeholders! How can we improve? The people here to teach are also here to learn’. (Meeting—F)	Both contributors and Cochrane team members reflected on the original experience positively, but agreed that disruption over the year had meant less engagement than planned. There was a mismatch in expected timescales: The CCMD facilitators indicated they were viewing the role of the VoE College as initiating a community contributing over years, whereas contributors may have expected more immediate opportunities to be available. Despite personal commitment, PPI was not prioritised as a core business deliverable for CCMD during this time.
Cognitive Participation	Mental health experience as an asset, bringing something different and essential into the academic process. ‘mental health gives you attributes that mean you have empathy and have connection … Even if people think oh I don't have that formal academic knowledge, they have that experience’. (T2—C2) Includes a range of expertise and skills, including those with academic backgrounds, health services backgrounds, expert by experience backgrounds ‘it's breaking down barriers—the assumed us and them of researchers and people with experience’. (Meeting—C) ‘Different experiences and backgrounds but all want something positive to come from lived experience and want to be a community of learners’. (Meeting—C) Less clear how could or should interact with the wider CCMD team.	Recognition that consumers bring unique and necessary knowledge, and also offer multiple (personal and professional) expertise. ‘They're the experts. They bring a lot of different skills, networks, they're such a strong group’. (T1—F) Recognised need to build a community—aiming to enrol contributors into partnership working with CCMD. ‘it's about building a critical mass of reviewers but it's also about building relationships’. (T1—F) ‘We know consumers can feel separate. We want them to feel part of Cochrane‘. (T1—F) Contributors valued the CCMD facilitators' soft skills, good communication and facilitation. ‘They weren't patronising, they were empathetic, they had fun but it was focused’. (T1—C1) ‘[researcher] was pivotal in making me feel comfortable joining’ (Meeting—C) ‘Supportive, fun and friendly team’. (Meeting—C) One member of the CCMD facilitators was a Consumer Reviewer and her sharing her experiences emphasised the need for lived experience and modelled the contribution that could be made. ‘She was inspirational. You think, if she can do it, I can do it’. (T2—C6) ‘Her story was touching, I could then relate this to real life’. (Meeting—C)	Ongoing activities were dependent on individuals to initiate—both individual members of the broader CCMD team being responsible for creating and offering opportunities, and individual contributors needing to be proactive in seeking opportunities themselves. This dependency on individual effort was challenging for both CCMD and some contributors to manage. ‘I haven't heard much. But I know the campus was shut down. I knew who to get in touch with, and how, if I wanted to. I felt that was up to me’. (T2—C6)
Collective Action	For some consumers, it was unclear if and how they would be contributing after the College. ‘I remember thinking this is cool, I love this, but where is it going? … I felt there was a hesitancy to say “we want you to do this”’. (T2—C4) ‘I would like encouragement to participate in more research, opportunities going forward’. (Meeting—C) For those involved, felt the College had prepared them well for the reviewing, although felt that peer examples or ‘buddies’ could help build confidence early on.	CCMD facilitators were anxious about appearing to expect too much time commitment from contributors. ‘Maybe we're over sensitive in mental health, but we worry a lot, we want to be mindful of them having lots to deal with’. (T2—F) Established mechanisms in CCMD for consumer review. Cochrane network and resources demonstrate organisational commitment to involvement. ‘Patients are number 1 on the dissemination checklist. They're core to what we want to do’. (T1 Meeting—F)	A lack of regular and routine opportunities meant that there was little interaction between the group (both CCMD facilitators and contributors) as a whole. ‘We've had a gulf where we haven't had the number of events we thought we thought we would, especially face‐to‐face. It falls through the gaps’. (T2—F) ‘I haven't used my learning since. So my confidence has gone down. Some kind of reminders, just to check in, would have been useful’. (T2—C10)
Reflexive Monitoring	Felt that facilitators were responsive to feedback ‘We've always felt we've been listened to’. (T1 Meeting—C) Gained confidence, enjoyment of learning something new and feeling of being part of a group. ‘It's been about connecting with people. Learning from everyone’. (Meeting—C)	At the start of each session, completed a carousel feedback activity to hear from contributors about their experiences of the prior workshop and the online modules, and take suggestions for improvement. ‘They were really open to constructive criticism, really receptive. You could see the modules change over time’. (T2—C1) The evaluation process itself was collective, and it was important that the final output reflected both research and consumer perspectives.	The lack of contact meant that opportunities to reflect on what had been achieved or what had been missed were not available. This led some contributors to reflect negatively on the original College, as the lack of sustained action meant they questioned the authenticity of the original engagement (undermining earlier Coherence). ‘I'm not sure in hindsight if I think it was worthwhile, if I haven't used that learning. What was it for?’ (T2—C1)

Abbreviations: CCMD, Cochrane Common Mental Disorders; NPT, normalisation process theory; PPI, patient and public involvement.

At T1 the evaluation found strong evidence of coherence and cognitive participation. These were entwined, with the need for lived experience input into reviews and evidence production, meaning the value of the lived experience contributors was integral. Importantly, this was a collective view of both the contributors and the Cochrane team members. The CCMD facilitators' explicit valuing of the contributor's viewpoint was key in supporting their engagement.

[researcher] having confidence in me gave me the confidence to think I could do it (Meeting—C)

The CCMD facilitators wanted the VoE College to enable gains for the contributors from their own point of view, to avoid becoming a self‐serving exercise of creating a reviewer cohort purely to meet their own needs. The evaluation of both coherence and cognitive participation indicated this was successful, as contributors discussed how the VoE College achieved several personal goals, enabling them to gain new skills, knowledge and perspectives, to gain a sense of contributing to something bigger than themselves and of importance to others experiencing mental health problems and to meet and work with like‐minded people.

You can teach an old dog new tricks! It changed how I look at things. It changed how I write, my style, to be clearer. It's refreshed me. (T2—C6)

The gain in knowledge was personally fulfilling, rather than being a researcher‐set criterion for contribution, particularly for those whose own educational or professional aspirations had been disrupted by their mental health condition. Their experiential knowledge was seen as fundamental to their contribution, with the learning experience enabling them to ensure such experiences were considered within academic work, rather than academic knowledge overruling their lived experience.

Rather than being a process at the end of the work, reflexive monitoring was a collective and ongoing activity through the College that was a significant contributor to the coherence and cognitive participation (as opposed to these being linear factors). The openness of the research team to learning from the contributors, through actively seeking their feedback on the process throughout, enacted the attitude of valuing and needing input from contributors.

It was noted that several consumers had mixed personal and professional experience of mental health, with some coming from clinical backgrounds and others working in academia or studying for academic qualifications. This was viewed as a strength, with the College welcoming the multiple viewpoints this offered. The contributors felt it was too simplistic to consider this a limitation in terms of reaching potential contributors without professional backgrounds, and suggested a phased approach:

Maybe these [the College participants] are the people interested in making research more accessible and down the line you can try to reach those who aren't interested. We're still one step removed from the research. (T2—C4)

Nevertheless, contributors did suggest that future iterations of the College could consider how to be more inclusive and diverse:

You need diversity of viewpoints. Is a tweet from the University of York going to attract people if they left school at 15, are they going to come onto campus? It's not about excluding people who have higher education, the question is what experience do you want. If you want people with lived experience of mental illness, how do you make it that everybody's welcome? (T1—C1)

This point aligned with prepandemic plans, which had included an ambition to collaborate with local partner organisations to enable the team to deliver face‐to‐face VoE Colleges which may reach participants from more diverse backgrounds.

For people with an academic professional background or role, the VoE College was valued as a space to talk more critically about research and to ask bigger questions about why research was needed, confirming the value of spaces where lived experience questions could have prominence:

At work you have professional boundaries, office politics. You see the pressures of the work, the time and cost it takes to change things. But it gets in the way of that raw ‘What are we doing this for again? Should we be doing this?’ (T2—C4)

One challenge reported was around whether self‐disclosure of mental health was expected or, by contrast, discouraged. Although the value of expertise through experience was clear overall, it was less clear if and how they were expected to bring personal experience of mental health into the VoE workshops, with one contributor describing it as ‘*the elephant in the room*’. The CCMD facilitators explained they had not wished to pressure any individuals into disclosure (although, during the preparation of this paper, it was raised that this could be interpreted as a patronising assumption). Other contributors however felt that they did not expect to discuss their own experiences, and cautioned that discussion of personal experience could be triggering for other contributors, or felt it was inherent in the VoE College that their mental health experience was why they were needed. Reflecting on this at T2, contributors suggested that activities could be explored which enabled discussion of mental health experiences more broadly, to establish lived experience as central but without pressure to self‐disclose.

‘Collective action' within this evaluation partly involved conceptualising the feasibility of the College itself, but notably, for the contributors, included the plans for action beyond the College. Collective action at T1 was considered strong by the CCMD facilitators, who pointed to the embedded mechanisms requiring involvement, and CCMD commitments to involvement. Some contributors at T1 expressed they were unclear exactly how things would work going forward, but felt this would become apparent with time.

This issue became more prominent during the T2 evaluation (Table 2, column 4). Disruption to CCMD due to COVID meant plans for ongoing engagement had suffered. Contributors accepted that progress had not been as intended, but there was an evident difference between those who felt able to proactively reach out to CCMD to seek opportunities and those who felt disappointed by the lack of clarity and outreach from CCMD. Where this disappointment occurred, it could serve to undermine the original coherence and cognitive participation, as it caused contributors to question the original CCMD commitment to working with consumers and brought into question whether their own commitment to upskilling had been worthwhile. This issue was explored further under RQ2.

### 3.2 RQ2. What recommendations can be made to support the sustainability and spread of the College?

1. *Sustainability*:

The presence within CCMD of explicit mechanisms for the requirement of consumer feedback was anticipated as a strength at T1, as it indicated established routes to contribution (in terms of collective action, the presence of embedded routines into which contributors could expect to fit). Coronavirus disease‐2019 (COVID‐19) significantly disrupted this, however, and in doing so suggests weaknesses in this approach. While consumer reviews were an established part of CCMD, there were not established routines for maintaining contact with consumers in‐between occasions when reviews were needed, nor routines for engaging with the cohort as a community, rather than with individuals. The responsibility for this engagement was with a single CCMD team member (J. H.), and was a responsibility outside of the core business of the review group.

The CCMD facilitators reflected that they had expected opportunities for involvement to arise organically and this was upended by COVID‐19 disruption. The CCMD facilitators acknowledged that if sustained engagement had been planned more specifically, it may have been less likely to be disrupted. Supporting engagement was also dependent on receiving further funding, which meant that gaps occurred when funding bids were unsuccessful. Other academic conditions, such as uncertainty over research contracts, also inhibited plans for engagement as it was not clear if individual CCMD facilitators would be in the post to support the work.

Facilitator anxiety about appearing to pressure contributors can mean they do not actively encourage contributions. Contributors warned that this anxiety can be interpreted as actively not wishing for them to be further involved. This had been raised at the T1 evaluation:

If you don't come back to us, we think it's us. We think you weren't happy with what we did before, or think you've lost confidence in us. (T1—C)

However, anxiety about imposing burdens was amplified during the COVID‐19 disruption. Reflecting on this at the end of the evaluation, both contributors and CCMD facilitators suggested that more openly sharing these respective anxieties (that contributors would be pressured or that contributions were not wanted) would be helpful, and informal spaces for catch‐ups and reflections could help achieve this. This suggested reflexive monitoring was needed as an ongoing mechanism to support continued coherence and cognitive participation.

Mindful of the time demands on individual CCMD facilitators, contributors also suggested digital ways to maintain contact, such as having a dedicated board on the CCMD website or sending a monthly email bulletin. Providing summaries of contributions made was also suggested as a way of reassuring those contributors who had not been directly involved that consumer involvement had not stopped, and might provide opportunities for consumers to respond and ask to be informed of similar opportunities.

Some contributors also expressed appreciation that the facilitators were concerned about the burden and emphasised the need for flexibility. It was apparent from their comments, however, that regular communication could also have reassured contributors that it was entirely acceptable to the CCMD facilitators to drop in and out of contributions.

It wasn't something I could cope with mentally at the time [after the College]. I haven't had the capacity. But I felt it was on my initiative. My concern was that you had all put a huge amount of work in and have I let you down. (T2—C6)

CCMD facilitators described planning for sustainability in terms of touchpoints (specific interactions with wider Cochrane or CCMD), to recognise that an absence of interactions or a one‐off negative experience could be detrimental and so there needed to be a long‐term plan for the ‘journey’, which would be transparent to contributors.

People's trust can be eroded by a negative next touchpoint on the journey. We should be thinking 12‐24 months ahead after the College, and share that plan with the contributors. (T2—F)

2. *Spread*:

At T1, contributors had questioned whether other Cochrane groups would be as open to learning from consumers as CCMD had been. They attributed this openness both to the individuals involved and to a participatory culture in mental health, which recognises the value of service user perspectives.

This was explored in more depth at T2 with the CCMD facilitators and the Consumer Engagement Officer. While acknowledging that an attitude of openness was required, it was felt that different groups across the network increasingly embraced this openness, coming from their own different participatory contexts. Similarly, they suggested that different groups would need to tailor their approach and the materials to their specific review topic and to the specific opportunities that would be available for consumers in their settings. This would be particularly important to allow cultural sensitivity, recognising that Cochrane is a global network and that approaches would need to vary across regions.

The CCMD facilitators reported that they would emphasise the need for a significant time to be spent on the College on the relationships with contributors and not just on preparing materials. This included work to support contributors to engage (in the *VoE* College, J. H. had met each contributor one‐to‐one initially to talk through what was involved) and to seek and respond to feedback throughout. Again, considering the importance of relationships, the CCMD facilitators emphasised the importance of soft skills and would encourage groups to consider whether they have this expertise within their own settings. They suggested that support to consider facilitation and communication needs could be provided through peer‐to‐peer ‘train the trainers’ sessions and through establishing communities of practice across Cochrane, suggesting that a best practice understanding was developing which could and should be shared. They also emphasised that any research team establishing a College should have a consumer member working with them, again indicating the fundamental importance of collaborative working to the approach.

Shadow existing set ups. Learn from best practice elsewhere, don't re‐discover the same challenges. It needs to be resourced, particularly to have someone involved with lived experience. (T2—F)

## 4 DISCUSSION

We report a longitudinal collective evaluation of the *VoE* College. The College was evaluated positively, but with sustainability following the initial College proving to be a challenge and, for some contributors, potentially undermining their initial enthusiasm. The collective evaluation enabled the CCMD facilitators and contributors to share these challenges, acknowledging anxieties on both sides (for CCMD, fear of appearing to pressure contributors, and for contributors, fear that CCMD did not want them to be involved), and propose ways to overcome these challenges in future.

Training in PPI has been contentious, but our evaluation suggests that upskilling can deliberately recognise and embrace experiential knowledge (rather than ‘professionalisation’ undermining the lived experience contribution) and can be experienced as collaborative partnership building when researchers are themselves open to learning from and with the contributors. This openness can be built into the process through actively seeking feedback, ensuring time within the training sessions for contributors to share their experiences of training itself with the facilitators or researchers, and through modifying materials or processes as a result to demonstrate that feedback has been acted upon. Including expert contributors with lived experience as part of the training and facilitation team can also emphasise the collective approach and serve to reassure and inspire new contributors.

Contributors considered a strength of the training to be that they could bring multiple roles, with several having an overlap of professional (either academic or clinical) and service user experience. The College explicitly recognised and invited individual expertise of the contributors, beyond only their mental health experience. Contributors can criticise PPI activity where they are expected to be ‘just a patient’ and only valued for their experience of a particular health condition and can value the opportunity to embrace the multiple identities, personal and professional, that individuals bring.^20^ This supports the need to move beyond simplistic binaries, of being either a patient or a professional, to richer and more accurate conceptualisations of contributors such as bringing different skill sets and multiple types of lived experience.

One consideration for future involvement is how to enable personal experience to be ‘in the room’ without pressure to personally disclose. Creative and interactive methods which use design and serious play are increasingly seen as valuable in co‐production to allow meaningful sharing and reflection in patient involvement.^21^ Within this study, one contributor co‐author had, consistent with such methods, previously used the creation of visual metaphors using sweets as a way to encourage expression of priorities and concerns. The College deliberately adopted a playful approach to teaching about systematic reviews, and it may be helpful to plan similar activities that enable reflective discussion of lived experiences without pressure to directly disclose.

The evaluation offers further evidence that authentic involvement is achieved relationally. An National Institute for Health Research review of involvement progress included Relationships as a key determinant of success in strengthening involvement^22^ alongside Reach, Refinement and Relevance. Based on our findings, a fifth addition may be Reciprocity, in which the mutual value for both contributors and facilitators/researchers and their responsibilities to each other in achieving that value is explicitly discussed and explored. In this evaluation, assumptions on each side (that contributors would not value opportunities to apply their learning but instead would feel pressured, and that facilitators did not value further input into work beyond the VoE College) undermined the actual Reciprocity of the VoE College, suggesting it should be the focus of discussion in future activities.

### 4.1 Implications for supporting sustainability

COVID‐19 meant unexpected levels of disruption occurred and continuity between the workshops and ongoing work by the group was lost. Nevertheless, the evaluation suggests two limitations to sustaining involvement which are indicative of systemic problems, not limited to the pandemic period.

First, sustainability should not be reliant on relationships with individuals in an organisation, who will inevitably be juggling multiple responsibilities. This poses a challenge for involvement when those relationships have been so crucial to initial engagement. More thought is required about how to foster a sense of ongoing community involvement which can provide broader support and encourage access to opportunities.

Second, more explicit planning of further involvement with mechanisms to proactively engage contributors is necessary, rather than relying on opportunities to arise or relying on contributors themselves to maintain contact. This again should not be assumed to be the responsibility of individuals, but requires organisational commitment.

In the present study, the facilitators reflected that they would now much more explicitly suggest that VoE College graduates become members of the Cochrane Consumer network, and would also seek local involvement networks to link the contributors with (e.g., within the University). They would also timetable specific catch‐up events. The contributors, who themselves acknowledged that CCMD members and researchers were time‐poor, suggested digital ways of keeping in contact, such as quarterly email bulletins that could report consumer activities that had been completed and either advertise new opportunities coming up or reassure consumers that opportunities would be advertised once available. One issue to be explored in the future is how to balance researcher anxiety about burdening contributors with a recognition that contributors will want proactive encouragement. Some contributors observed that the anxiety could be considered condescending, underestimating the ability of the contributors to assess their involvement and communicate their wishes, and thereby inadvertently leading to paternalistic assumptions. It may also be helpful to explore in further work how different contributor experiences or backgrounds can impact on whether they feel comfortable to reach out directly, for example, recognising that different professional backgrounds and also different mental health service experiences may require different support.

The disruption to sustained engagement after the College demonstrates two risks to involvement:

First, while the challenges of building and sustaining relationships in the context of short‐term contracts and uncertain project funding is recognised, our evaluation demonstrates how these uncertainties can not only limit future involvement but can undermine the initial investments made. We hope that this adds additional weight to the need for sustainability to be seriously considered, as it can have retroactive effects on previously positive experiences.

Second, the disruption to intended plans, despite the initial commitment and time investment of CCMD individuals, indicates the fragility of the organisational commitment to PPI, which relied on individual time, and was not prioritised as core business. Organisational expressions of commitment to consumer involvement need to be matched with operational commitments and practical staff support which embed regular opportunities and support ongoing engagement as part of overall network activity. However, rather than only ensuring ongoing contributions, there is a need to commit to ongoing reflection. Bryant et al.,^10^ in their evaluation of collaborative involvement, reported the importance of creating space and making time, to reflect and to renegotiate roles and understanding, especially when circumstances change. This time and space can be one of the most challenging things to prioritise in academic settings, but the present evaluation demonstrates how essential it may be.

### 4.2 Implications for supporting spread

CCMD were optimistic that with adequate resourcing the VoE College process could be adopted by other research groups to help build or initiate their own expert consumer cohorts and partnerships. They described a ‘core and custom’‐style approach to spread, which would encourage different groups to tailor their content and use their own participatory ways of working (customisation), but would establish that time spent building relationships and a two‐way approach that sought feedback throughout were critical mechanisms (core). This is consistent with work emphasising the soft skills of researchers as essential to involvement,^23^, ^24^ and therefore the need for organisations that express commitment to involvement to give due attention to recognising, supporting and developing soft skills in the workforce. Far more than being ‘nice to have’, these skills were critical to engaging contributors and to the perceived effectiveness of the training. This will require, however, a recognition that such skills are of value. The soft skills required of consumers/public contributors themselves have been discussed,^23^ and accounts of PPI as told by researchers involved have emphasised the interpersonal and communication skills required (e.g., https://healthtalk.org/patient-public-involvement-researchers/Skills-needed-for-involvement). Williams et al.^25^ have pointed out that addressing a perceived skill deficit around interpersonal working in co‐production will require recognition of how historically these skills, and those who hold them, have been undervalued in academia to date. Some developments in the sector suggest there is willingness to re‐evaluate this, for example, the adoption by UK Research and Innovation of the researcher resumé instead of the traditional academic curriculum vitae, which asks about supporting the development of others and building relationships.

It was noted that the VoE College facilitation team had been composed of Cochrane professionals with operational experience and expertise in facilitation, and it is unclear currently whether a team of exclusively academic professionals (e.g., researchers) could deliver the VoE College approach without relevant prior training or experience. The additional resource and time needed to enable relational working also need to be anticipated.^26^

As well as the spread of the approach to other review groups, both contributors and facilitators discussed spread in terms of further tailoring the College to more diverse groups within mental health. Involving ‘expert’ consumer reviewers who have themselves been on a journey of understanding reviews was considered to be key to anticipating these different needs, with a phased approach that worked with existing contributors to develop further reach and inclusive opportunities. We also reflected that making the College more inclusive and accessible would require us to revisit some of our decisions about reimbursement. While we originally planned to only provide out‐of‐pocket expenses for attendees of the course, we reflected on whether this may prohibit some groups from attending, and therefore fully supporting inclusivity in capacity building in the future should also provide compensation for the time given.

### 4.3 Implications for evaluation

The study demonstrates the importance of longitudinal evaluation of involvement activity. This should include opportunities throughout to engage in shared sense‐making, tackle assumptions and address concerns. Learning from each other within the College itself was an integral mechanism throughout that established two‐way respect and learning, not simply a retrospective or summative reflection. The longitudinal findings suggest this ongoing reflection is likely to be crucial at longer time scales. Opportunities not only for direct contributions but also for opportunities to discuss and understand the need for contribution and how this can fit into changing circumstances should therefore be built into sustainability plans. This ongoing communication proved a challenge even during the preparation of this paper, however, with long delays between contacts with contributors despite the researcher's best intentions and awareness of the problem. This further emphasises that addressing this problem requires organisational commitment for implementation, to support researchers to achieve best practice.

The collaborative evaluation was helpful in engaging both facilitators and contributors in suggesting solutions and tackling assumptions made. This enabled us to move beyond diagnosis of problems, to propose solutions for the future. The collaborative evaluation was also considered most appropriate by all (facilitators and contributors) as it appropriately reflected the collaborative journey of the College itself. Blending this collaborative approach with a theoretical framework (NPT) enabled us to ensure we attended to broad issues at an organisational and operational level, but rooted these in the personal learning and concerns of both the facilitators and contributors. This enabled us to produce recommendations for the future which are both conceptually and experientially informed. The NPT framework was considered a valid and useful organising framework by the contributor co‐authors and the CCMD facilitators that were unfamiliar with it, but a barrier to use and reporting was the very academic terminology.

The paper is co‐authored by contributors. This was crucial in ensuring that we presented a collective reflection, rather than the facilitators' perspectives alone, to ensure that evaluation of PPI activity does not itself become ‘for and about’, rather than occurring with the active involvement of contributors themselves. The contributor input changed the manuscript in the following ways:

1. Questioning the assumptions behind the facilitator's perception of responsibility for contributors which could be interpreted as patronising, suggesting a need for more open dialogue about this in future involvement.
2. While acknowledging overall findings, wishing to nevertheless emphasise the diversity of responses, for example, that not all individuals felt the same way about the online modules or about the level of the material. Rather than disagreeing with the summary findings, contributors wished to acknowledge any summary would neglect individual variation to an extent. Recognising that one size does not fit all with training, and being sure to not expect blanket approval or dislike of training components, is an important message from this.
3. While acknowledging that NPT constructs fitted the feedback, contributors suggested there needed to be critical reflection on the framework itself, and the off‐putting terminology was considered the key limitation. The framework was chosen to help structure our evaluation with transferable learning as an outcome, to identify issues that are known to typically impact whether an activity—in this case, the College—is successful or not. Although NPT was initially presented as an option for framing the evaluation and proceeded based on consumer agreement, the choice of this particular framework was from an academic author (S. K.) who had previously used the theory successfully in PPI discussions with public contributors.^27^ In this previous instance, the theory was presented to the PPI group as it was already the chosen framework for the overall implementation evaluation of the trial. There is therefore a gap regarding genuine collaborative identification of theoretical frameworks. We also acknowledge that there has been criticism of bringing frameworks designed to evaluate interventions into PPI evaluation,^9^ although we tried to be mindful that we were not evaluating the PPI endeavour itself as if it could be successful or not, but the process of the College being delivered and facilitated. We recommend in future that authors consider whether theory can be expanded, revised or developed anew with contributors themselves, bringing the crucial partnership of ‘with, not about’ into the use of theory itself (and indeed potentially into the decision about whether theory is appropriate and helpful in collaborative work, or whether it primarily serves academic and publishing requirements). Collaborative development of theory for understanding PPI has been demonstrated previously^28^ and research with lived experience perspectives has been shown to generate novel theoretical angles.^29^

## 5 CONCLUSION

The collective evaluation demonstrates that, contrary to opinions that training can threaten the lived experience of contributors, training can be positively experienced as recognising the value contributors bring. This is achieved through openness from those delivering the training to two‐way learning and through relational support. Networks and organisations seeking to involve consumers should recognise that such ‘soft skills’ are crucial mechanisms of engagement. Sustainability of involvement beyond initial activities was found to be the most significant challenge encountered and could threaten initially positive experiences. Prioritising sustained communication (including adequately resourcing this and exploring how it can be an organisational, rather than individual responsibility) and providing opportunities to revisit roles and expectations are recommended for future involvement work.

## AUTHOR CONTRIBUTIONS

Jessica Hendon and Karen Morley designed and delivered the College, in collaboration with other Cochrane professionals. Sarah Knowles and Jessica Hendon designed the evaluation. Sarah Knowles wrote the first draft of the paper. All co‐authors contributed to the evaluation and reviewed and contributed to the final paper.

## CONFLICT OF INTEREST STATEMENT

The authors declare no conflict of interest.

## ETHICS STATEMENT

Ethical approval is not required for public involvement activity. The contributors and researcher/consumer members of CCMD engaged in the evaluation collaboratively as partners collectively providing and reflecting on feedback, rather than data being collected by or about contributors (or the facilitators) separately. We sought expert guidance from the Chair of the York Health Sciences Research Ethics Committee, who confirmed that the evaluation was itself a public involvement activity, rather than a research activity conducted on or about participants, and therefore did not require Research Ethics Committee approval.

## Supporting information

Supporting information.

## Data Availability

Materials used in the College itself are available online. Data sharing is not applicable to this article as no data sets were generated or analysed during the current study.

## Footnotes

* Within the manuscript, we use the term ‘contributors’ to refer to people with lived experience of mental health problems (e.g., as service users or carers). We use the term ‘consumers’ when we are referring specifically to contributor activity or membership within the Cochrane organisation.
