# Supplementary material for: Collaborative evaluation of a pilot involvement opportunity: Cochrane Common Mental Disorders Voice of Experience College
Source: Health Expect. 2023 Aug 15;26(6):2428–40. doi: 10.1111/hex.13835 (PMC10632641; doi:10.1111/hex.13835)
Supplement: Supplementary file 1 — Supporting information. [file HEX-26--s001.docx]

Appendix1 – Learning outcomes and aims for the three VoE College Workshops

|  | **Workshop 1** | **Workshop 2** | **Workshop 3** |
| --- | --- | --- | --- |
| **Title** | Discovery | Understanding | Applying |
| **Aims** | Forming as a group. Preparing for evaluation and feedback from the group. Overview of the importance of evidence, EBM and Cochrane. | Receive evaluation and feedback. In greater detail, explore and understanding Cochrane evidence and how to use it.  (Use feedback to plan Workshop 3) | Feedback on Cochrane Evidence Essentials. Learn how to find and use evidence for your own shared decision making and how shared decision making looks on the ground. Feedback on the course as a whole. |
| **Learning Outcomes** | Be able to recognise misleading health claims in the media  Understand the ‘three components’ model of Evidence Based Medicine  Be familiar with shared decision making and understand some of the issues related to it  Start to understand the role of Cochrane and the quality evidence it publishes | More understanding of:  Clinical trials and some of their important features and shortcomings  What a Cochrane Systematic Review does  Forest plots  The role of Cochrane and the quality evidence it publishes | More understanding of:  What the Cochrane Library is, and how to search for Cochrane evidence  The process of shared decision making, the different ways in which Cochrane evidence might be used  Where to find resources to help in making healthcare choices  The role of Cochrane consumers in co-producing evidence  How you can get involved in Cochrane |

.

**Appendix 2 – Discussion guide for T2 discussions**

**Topic Guide:**

1. Have you been involved in Cochrane activities since the college?
- do you think the college prepared you for these? Is there anything that could have helped you more?
2. Have you been involved in any further training eg. Blogging workshop?
3. Have you been involved in any other research or health work?
- has the college work helped you to be involved? Eg. Greater confidence, familiarity with research processes, understanding of evidence
4. Have there been any other times or situations where you have used the learning from the college? Eg. As a user of health information (as a patient/carer/volunteer)?
5. Reflecting over the past year, are there things we could have done more of to stay in contact or offer opportunities?
- how could we best keep in touch with people? What kind of updates would be of interest?
- What kind of opportunities could we offer? Eg. Further training, catch up events, volunteering for specific or more Cochrane activities.

6. Looking back at the initial evaluation findings, do you think this captures the key points? Is there anything that you have changed your mind about, or additional reflections looking back?
